# Pro‐inflammatory cytokine IL‐6 regulates LMO4 expression in psoriatic keratinocytes via AKT/STAT3 pathway

**DOI:** 10.1002/iid3.1104

**Published:** 2023-12-06

**Authors:** Zhenzhen Tu, Wei Wei, Qiantong Xiang, Wenwen Wang, Siping Zhang, Haisheng Zhou

**Affiliations:** ^1^ Department of Immunology, School of Basic Medical Sciences Anhui Medical University Hefei China; ^2^ Department of Dermatology Affiliated Provincial Hospital of Anhui Medical University Hefei China; ^3^ Department of Dermatology Second People's Hospital of Hefei Affiliated of Anhui Medical University Hefei China; ^4^ Department of Biochemistry and Molecular Biology, School of Basic Medical Sciences Anhui Medical University Hefei China; ^5^ The Center for Scientific Research of Anhui Medical University Hefei China; ^6^ The Institute of Dermatology Anhui Medical University Hefei China

**Keywords:** AKT, interleukin‐6, LMO4, psoriasis, STAT3

## Abstract

The transcription factor LIM‐only protein 4 (LMO4) is overexpressed in the psoriatic epidermis and regulates keratinocyte proliferation and differentiation. High LMO4 expression levels are induced by interleukin‐23 (IL‐23) to activate the AKT/STAT3 signaling pathway. Interleukin‐6 (IL‐6) is mainly involved in regulating T cell functions and development in patients with psoriasis. However, whether LMO4 expression is regulated by IL‐6 remains unclear. Therefore, the purpose of this study is to explore the role and molecular mechanisms of IL‐6 in regulating LMO4 expression. The interleukin‐6 (IL‐6) levels in human plasma were determined using a chemiluminescence immunoassay system. A psoriasis‐like mouse model was established using imiquimod induction. Epidermal keratinocytes (HaCaT) were cultured in defined keratinocyte‐serum‐free medium and stimulated by IL‐6 alone or with inhibitors. The proteins of interest were detected using western blot analysis, immunofluorescence, and immunohistochemistry. The 5‐ethynyl‐2′‐deoxyuridine assay was used to detect cell proliferation. The results revealed that IL‐6 levels were markedly increased in the plasma of patients with psoriasis, compared to healthy control. The high expression of LMO4 was consistent with high levels of IL‐6, p‐AKT, and p‐STAT3 in the lesions of both psoriasis patients and imiquimod‐induced psoriasis‐like mice. IL‐6 activates the AKT/STAT3 signaling pathway, followed by LMO4 high‐expression in HaCaT cells. IL‐6 induces HaCaT proliferation and differentiation via AKT/STAT3 signaling pathway activation. We think that the high expression of LMO4 in psoriatic keratinocytes requires IL‐6 to activate the AKT/STAT3 signaling pathway and leads to epidermal keratinocytes abnormal proliferation and differentiation.

## 1 INTRODUCTION

Psoriasis is a common hyperplastic chronic inflammatory skin disease characterized by persistent inflammation resulting in the parakeratosis, abnormal differentiation, and hyperplasia of epidermal keratinocytes.^1^, ^2^, ^3^ The inflammatory milieu activates signaling pathways to induce keratinocyte proliferation and differentiation depending on different nuclear transcription factors, such as signal transducer and activator of transcription (STAT)‐1,^4^ STAT‐3,^5^, ^6^ nuclear factor‐κB (NF‐κB),^7^ MAF bZIP transcription factor B,^8^ interferon regulated factor‐1 (IRF‐1),^9^ ovo‐like transcriptional repressor 1,^10^, ^11^ grainyhead‐like (GRHL),^12^ and LIM‐only protein 4 (LMO4). LMO4, a member of the LMO family, predominantly regulates the proliferation and differentiation of epithelial cells during embryogenesis.^13^ Previous studies reported that LMO4 is mainly expressed in the basal cell layer of healthy epidermis.^14^, ^15^, ^16^ Although a significant effect of *Lmo4* deletion on epidermal development has been reported, defects in epidermal terminal differentiation were more severe in both *Get‐1* ^‐/‐^ *Lmo4* ^‐/‐^ mice than in *Get‐1* ^‐/‐^ mice, which exhibited significant dyskeratosis. Therefore, LMO4 may interact with Get‐1, which is involved in epidermal barrier formation and the regulation of epidermal terminal differentiation.^14^, ^17^, ^18^, ^19^ Importantly, we previously found that LMO4 overexpression in the psoriatic epidermis leads to keratinocyte hyperproliferation and prosoplasia. A constitutively high expression of LMO4 in psoriatic keratinocytes is required to activate the AKT/STAT3 signaling pathway, which is triggered by interleukin (IL)‐23.^16^

The upregulation of other cytokines, such as IL‐6, IL‐12, IL‐22, IL‐29, IL‐36, IL‐1 β, and tumor necrosis factor‐α, is also involved in the pathogenesis of psoriasis.^20^, ^21^, ^22^, ^23^, ^24^, ^25^ Among these cytokines, IL‐6 is a pleiotropic cytokine involved in epithelial and immune cell angiogenesis, proliferation, and differentiation.^26^, ^27^, ^28^, ^29^ Owing to its excessive activation and dysregulation of receptor signaling in T cells, IL‐6 is involved in the pathogenesis of psoriasis.^30^, ^31^, ^32^, ^33^ IL‐6 contributes to the differentiation and maintenance of T helper (Th) 17 cell development and function and inhibits regulatory T cell (Treg) differentiation.^31^, ^34^ In contrast, an increased IL‐6 level in psoriatic lesions is involved in regulating keratinocyte proliferation and differentiation.^35^ However, little is known regarding whether LMO4 overexpression in psoriatic keratinocytes is required for IL‐6 induction.

Therefore, we hypothesized that LMO4 overexpression in the psoriatic epidermis may be caused by IL‐6 induction, resulting in psoriatic keratinocyte hyperproliferation and mis‐differentiation. We showed that IL‐6 levels are significantly increased in the plasma of psoriasis patients versus healthy donors. LMO4 overexpression was consistent with increased IL‐6 levels in both human psoriatic lesions and imiquimod‐induced psoriasiform dermatitis. IL‐6 induces LMO4 expression by activating the AKT/STAT3 signaling pathway to regulate keratinocyte proliferation and differentiation. Thus, the IL‐6/AKT/STAT3/LMO4 pathway is a potential therapeutic target for psoriasis treatment.

## 2 MATERIALS AND METHODS

### 2.1 Clinical specimens

Skin biopsies and peripheral blood samples were obtained from outpatients at the Affiliated Anhui Provincial Hospital of the Anhui Medical University. Informed consent for the experiments was not required because Chinese laws consider the human tissues and plasma left from surgery or clinical examination discarded materials. The use of pathological specimens and the review of all pertinent patient records were approved by the Ethics Review Board of the Affiliated Anhui Provincial Hospital of the Anhui Medical University. We've acquired psoriatic lesions and surrounding tissues from 6 patients with psoriasis, and foreskin from 4 healthy donors. These specimens were fixed in 4% paraformaldehyde, embedded in paraffin, and sectioned. Human plasma was frozen at −80℃ for subsequent experiments. Interleukin‐6 (IL‐6) levels in plasma of 18 donors and 26 patients with psoriasis were determined using a chemiluminescence immunoassay system (Immunlite 1000; Siemens Healthineers).

### 2.2 Animals model

A total of 12 BALB/c mice (6‐8‐week old male) were purchased from the Center for Laboratory Animal Science of Anhui Medical University. The mice were housed at a constant temperature and humidity with a 12‐h light/dark cycle and allowed a free diet. Twelve mice were randomly and evenly divided into control and imiquimod groups. A 2 × 2 cm^2^ area was shaved on the back of each mouse 48 h before experiment. A daily dose of 50 mg imiquimod cream (5% IMQ; MedShine) or Vaseline (V8230; Solarbio) was used on the back of each mouse for six consecutive days.^36^, ^37^ On the sixth day, the mice were killed, and the skin of the shaved backs was fixed with 4% paraformaldehyde at least 24 h, dehydrated with a series of graded alcohol, embedded with paraffin, and sectioned using HistoCore AUTOCUT (Lecia Biosystems). All animal experiments were performed in compliance with the Guide for the Care and Use of Laboratory Animals and the ethical guidelines of Anhui Medical University (NO.20200007). All procedures were performed in accordance with the China Council on Animal Care and Use guidelines.

### 2.3 Cell culture

Human immortalized epidermal keratinocytes (HaCaT cells) were obtained from the National Collection of Authenticated Cell Cultures. The HaCaT cells were cultured in defined keratinocyte serum‐free medium (d‐KSFM) containing d‐KSFM growth supplements (Gibco; Cat#10744019). HaCaT cells used to analyze the effects of 15 ng/mL IL‐6 (Sigma Aldrich; Cat#SRP3096) alone or in combination with inhibitors (JAK2 inhibitor: AG490, Cat#HY‐12000; AKT inhibitor: MK‐2206 dihydrochloride, Cat#HY‐10358; STAT3 inhibitor: NSC 74859, Cat#HY‐154146; MedChemExpress) on cell proliferation were cultured in Dulbecco's modified Eagle's medium (Hyclone Cat#SH30022.01) supplemented with 10% fetal bovine serum (ExCell Bio; Cat#FSP500). All the cells were cultured at 37℃ in a humidified incubator with 5% CO_2_.

### 2.4 5‐Ethynyl‐2′‐deoxyuridine assay

HaCaT cell proliferation was detected using a 5‐ethynyl‐2′‐deoxyuridine (EdU) Cell Proliferation Kit with Alexa Fluor 488(Cellorlab; Cat#CX002). Edu, structurally similar to the natural nucleoside, can be coupled via click chemistry. The small sized dye‐azide allows for efficient EdU detection upon incorporation.^38^, ^39^ HaCaT cells were treated with phosphate‐buffered saline (PBS), IL‐6 (15 ng/mL), or IL‐6 (15 ng/mL) plus inhibitors (AG490: 10 μM; MK‐2206: 10 μM; NSC 74859: 80 μM) for 21 h, and then incubated with 10 μM EdU for 3 h in a CO_2_ incubator at 37℃. Cells were collected using 0.25% trypsin, fixed with 4% paraformaldehyde, permeabilized with 0.3% Triton X‐100, and stained with a click reaction solution protected from light for 30 min. Then, cell reproduction was analyzed using flow cytometry (Beckman Coulter; CytoFlex). Cells were washed with FACS buffer (3% bovine serum albumin in 1 × PBS) throughout the experiment.

### 2.5 Western blot analysis

Total protein was extracted from HaCaT cells using RIPA lysis buffer (Biosharp; Cat#BL504A), and the total concentration of sample proteins were measured using the BCA Protein Assay Kit (Biosharp; Cat#BL521A). The total protein was separated using FuturePAGE (ACE Bio; Cat#ET15420Gel), transferred onto a polyvinylidene fluoride membrane (Millipore; Cat#IPVH00010), and blocked with 5% nonfat milk in PBST.

The following primary antibodies were used for western blot analysis: anti‐Akt (4691, 1:1000, CST), anti phospho‐AKT (R22961, 1:1000, ZENBIO), anticytokeratin1 (ab93652, 1:2000, abcam), anticytokeratin5 (ab53121, 1:2000, abcam), anti‐involucrin (R381850, 1:1000, ZENBIO), anti‐LMO4 (R389382, 1:1000, ZENBIO), anti‐STAT3 (9139, 1:2000, CST), antiphospho‐STAT3 (381552, 1:1000, ZENBIO). The membrane was subsequently incubated with the primary antibody at 4℃ overnight, followed by incubation with the secondary antibody goat antirabbit IgG (7074, 1:5000, CST) or horse antimouse IgG (7076, 1:5000, CST) at room temperature for 2 h. Finally, chemiluminescence was detected using WesternBright ECL horseradish peroxidase (HRP) substrate (Advansta; Cat#K‐12045).

### 2.6 Immunofluorescence staining analysis

HaCaT cells were seeded on glass coverslips and treated with IL‐6 or PBS for 3 days. The culture medium was discarded and the HaCaT cells were fixed, permeabilized with 0.3% Triton X‐100, and blocked with 3% bovine serum albumin. Next, the cells were incubated overnight at 4°C with the primary antibodies. The following primary antibodies were used: anti‐cytokeratin1 (ab93652, 1:100, abcam), anticytokeratin5 (ab53121, 1:200, abcam), anti‐involucrin (sc‐21748, 1:50, Santa Cruz) and anti‐LMO4 (SAB1404596, 1:100, Sigma‐Aldrich). Subsequently, the cells were incubated with appropriate secondary antibodies (4408, 1:200; 4414, 1:200; CST) at 37℃. Images were captured using an TissueFAXS Plus software (TissueGnostics Gmbh, TissueFAXS Plus S).

### 2.7 Histopathological and immunohistochemical analysis

Paraffin‐embedded tissue sections were mounted on glass slides, deparaffinized with xylene and hydrated with a series of graded alcohol. Then sections were stained with hematoxylin and eosin (H&E) to observe the histological structure, and target proteins were detected using immunohistochemistry (IHC). IHC was performed using a non‐biotin assay kit (PV‐9000; ZSGB‐BIO). Briefly, after the slices were deparaffinized, antigen retrieval was performed using citrate buffer, cool to room temperature. Peroxidase activity within the sections was blocked with 3% hydrogen peroxide (reagent 1, PV‐9000), and the slides were then incubated at 4℃ overnight with primary antibodies. The following primary antibodies were used for IHC analysis: antiphospho‐Akt (4060; 1:100; CST), antiphospho‐Stat3 (4113; 1:100; CST), anti‐LMO4 (SAB1404596; 1:450; Sigma‐Aldrich), anti‐IL‐6 (R1412‐2; 1:200; HUABIO). After being washed with PBS, the sections were incubated with a reaction‐enhancing solution (reagent 2, PV‐9000) and HRP‐conjugated goat antimouse/rabbit IgG polymer (reagent 3, PV‐9000). Finally, the tissues were visualized using freshly prepared diaminobenzidine solution (ZLI‐9018; 1:20; ZSGB‐BIO). After the proteins were visualized, the nuclei were stained with hematoxylin. Digital images were captured using an TissueFAXS Plus software.

### 2.8 Statistical analysis

**Figure 1:**
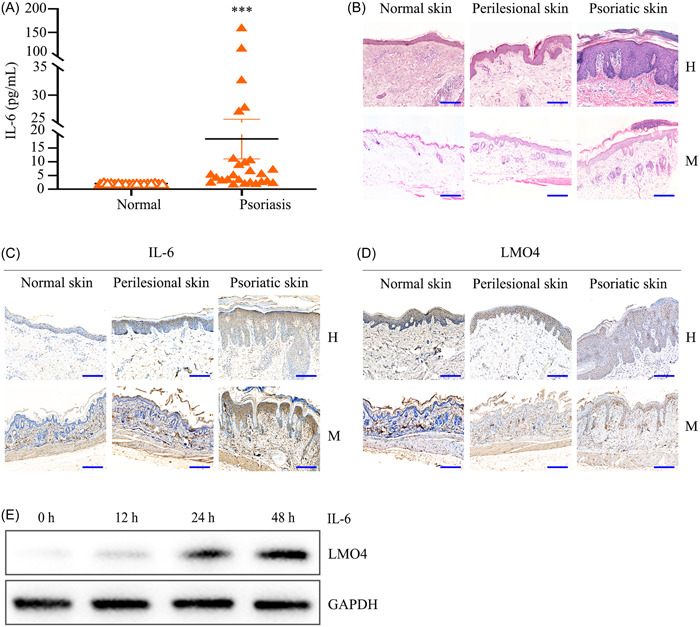
IL‐6 upregulates LMO4 expression in psoriatic keratinocytes. (A) Plasma IL‐6 levels in patients with psoriasis versus healthy controls using a chemiluminescence immunoassay system. (B) Hematoxylin and eosin staining of human and mouse skin tissue sections. (C) Histopathological staining of IL‐6 in skin sections from healthy controls and psoriatic lesions of human and mouse. (D) Immunohistochemical analysis of LMO4 in human and mouse skin sections. (E) Immunoblotting analysis of LMO4 expression induced by IL‐6 (15 ng/mL) in epidermal keratinocytes HaCaT cells. Scale bars = 20 μm. H, human skin tissues from a healthy control versus a patient with psoriasis; M, tissue sections of the imiquimod‐induced psoriasis‐like dermatitis and normal mouse skin; LMO4, LIM domain only 4; IL‐6, interleukin (IL)‐6.

All experiments set up at least three duplicate wells. The statistical analysis was performed with GraphPad Prism 8 (version 8.3). Values are expressed as mean ± standard error of the mean (SEM). Mann‐Whitney U was used in Figure 1A to compare the difference between normal and psoriasis group. Unpaired two‐sided Student's *t*‐test was used in Figure 3B to compare the proliferation between control and IL‐6 group. A one‐way analysis of variance (ANOVA) was used in Figure 4B to compare the proliferation between groups. Statistical significance was set at *p* < .05.

## 3 RESULTS

### 3.1 Expression profile of IL‐6 and LMO4 in clinical samples

A chemiluminescence immunoassay was used to detect plasma levels of IL‐6 in healthy donors and patients with psoriasis. IL‐6 levels were significantly increased in the plasma of patients with psoriasis versus healthy controls (Figure 1A) (***p < .001). Paraffin‐embedded skin specimens from patients with psoriasis and imiquimod‐induced psoriasis‐like mice were collected to determine the expression profiles of IL‐6 in the epidermal tissues. An H&E staining analysis of psoriatic lesions showed classic characteristics including epidermal hyperplasia, parakeratosis, acanthosis, elevated dermal papilla, and inflammatory infiltration. The pathological changes in the psoriasis‐like murine skin resembled those in patients with psoriasis (Figure 1B).

To detect the expression profiles of IL‐6 and LMO4 in the skin tissues, paraffin‐embedded skin specimens were subjected to immunohistochemical analysis. As shown in Figure 1C and 1D, IL‐6 was expressed at lower levels in healthy skin and LMO4 was expressed only in the basal layer keratinocytes of the epidermis. Increased IL‐6 expression was observed in every layer of the hyperplastic epidermis. LMO4 was highly expressed in the dermis and epidermis, consistent with IL‐6 expression in perilesional and psoriatic lesions, and was only expressed in keratinocytes located in the basal layer of the epidermis of healthy skin.

Furthermore, to investigate whether IL‐6 induces LMO4 expression in keratinocytes, HaCaT cells were treated with 15 ng/mL IL‐6 for 0, 12, 24, and 48 h. A western blot analysis analysis showed that LMO4 expression was induced by IL‐6 in a time‐dependent manner (Figure 1E).

### 3.2 IL‐6‐induced LMO4 expression in keratinocytes requires AKT/STAT3 signaling pathway activation

**Figure 2:**
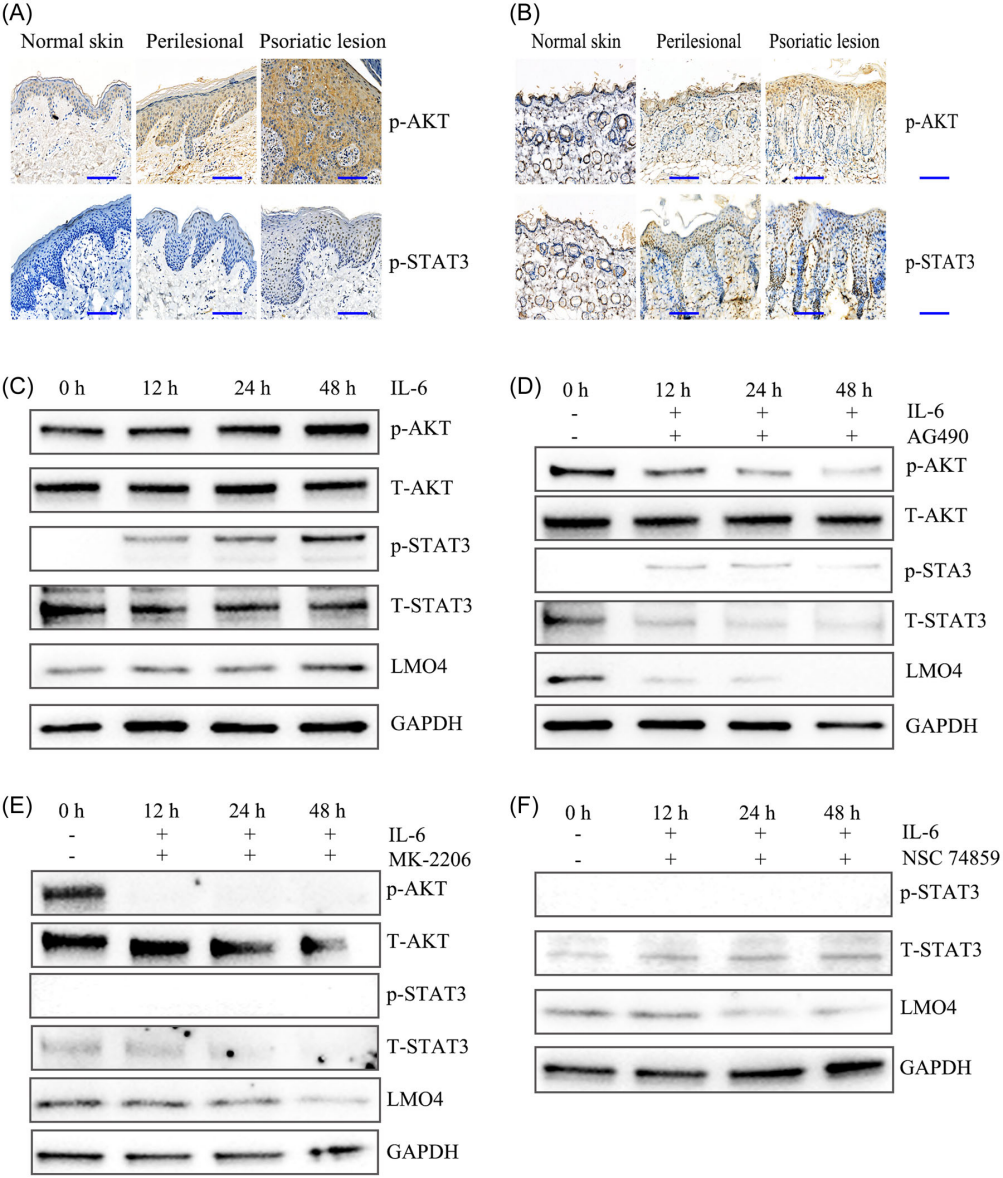
IL‐6‐induced LMO4 expression via AKT/STAT3 signaling pathway activation. (A) Immunohistochemical staining of p‐AKT and p‐STAT3 in human normal epidermis, perilesional tissue, and psoriatic epidermis. (B) Histopathological staining of IL‐6, p‐AKT, and p‐STAT3 in mouse normal epidermis, perilesional tissue, and imiquimode‐induced psoriasis‐like dermatitis. Scale bars = 20 μM. Detection of LMO4 expression induced by IL‐6 or after inhibition AKT and STAT3 using western blot analysis (c‐f). (C) The LMO4 expression induced by IL‐6 (15 ng/mL) was consistent with the increased p‐AKT and p‐STAT3 expression in HaCaT cells. (D) LMO4 expression was decreased after inhibition of AKT and STAT activation in HaCaT cells exposed to IL‐6 and JAK2 inhibitor (AG490). (E) The AKT inhibitor MK‐2206 dramatically reduced p‐STAT3, STAT3, and LMO4 expressions. (F) The STAT3 inhibitor NSC 74859 effectively inhibited STAT3 expression and activation. AKT, Protein kinase B; p‐AKT, phospho‐AKT; STAT3, Signal transducer and activator of transcription 3; p‐STAT3, phospho‐STAT3; JAK2, Janus kinase 2.

To investigate whether IL‐6‐induced LMO4 expression is required for AKT/STAT3 signaling pathway activation within psoriatic lesions, immunohistochemical staining was performed to examine the p‐AKT and p‐STAT3 levels. The p‐Akt and p‐Stat3 levels were markedly increased in the human psoriatic epidermis versus the perilesional tissues and normal epidermis (Figure 2A). Consistent with human psoriatic lesions, the AKT/TAT3 signaling pathway was also abnormally activated in the psoriasiform lesions of mice (Figure 2B).

To determine whether IL‐6 regulates LMO4 expression via AKT/STAT3 signaling pathway activation in keratinocytes, HaCaT cells were cultured for 0, 12, 24, and 48 h in d‐KSFM containing IL‐6. Immunoblotting analysis showed a significant increase in p‐AKT and p‐STAT3 levels in HaCaT cells after exposure to IL‐6, consistent with the upregulation of LMO4 expression (Figure 2C). And then HaCaT cells were then treated with the JAK2 inhibitor (10 μM AG490) or AKT inhibitor (10 μM MK‐2206) plus IL‐6 to block the signaling pathway, and we found that LMO4 expression was markedly decreased, consistent with the inhibition of AKT and STAT3 activities (Figure 2D,E). Furthermore, a substantial decrease in p‐STAT3 levels and LMO4 expression were observed after exposure to a STAT3 inhibitor (80 μM NSC 74859) (Figure 2F).

### 3.3 IL‐6‐induced LMO4 expression regulates keratinocytes proliferation and differentiation via AKT/STAT3 pathway activation

**Figure 3:**
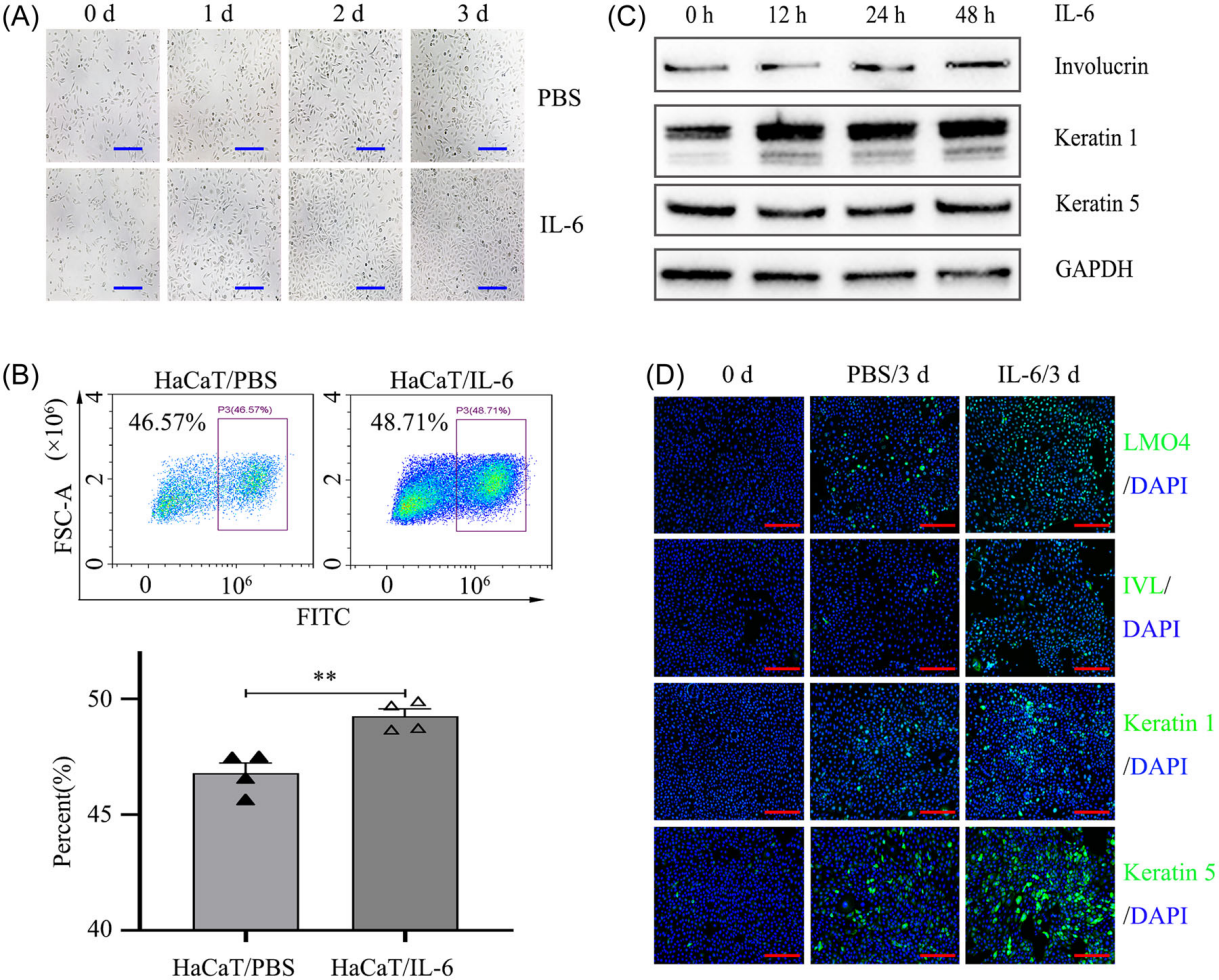
IL‐6 upregulates LMO4 expression and promotes HaCaT cell proliferation and differentiation. (A) Morphological changes in HaCaT cells after stimulation with IL‐6 (15 ng/mL) for 0, 1, 2, or 3 days. Scale bars = 50 μM. (B) EdU incorporation analysis of proliferation of HaCaT cells treated with PBS or IL‐6. The data represent the mean ± SEM of four independent experiments. **p < .01 versus HaCaT/PBS. (C) Immunoblotting analysis of involucrin, keratin 1, and keratin 5 expressions in HaCaT cells treated with IL‐6. (D) Immunofluorescence analysis expression of LMO4 (Green), differentiation markers K1 (Green), K5 (Green) and IVL (Green) in HaCaT cells with or without IL‐6 treatment. Scale bars = 20 μM. EdU, 5‐ethynyl‐2′‐deoxyuridine; IVL, involucrin; K1, keratin 1; K5, keratin 5; PBS, phosphate‐buffered saline.

To understand the roles of IL‐6‐induced LMO4 highexpression in regulating keratinocyte proliferation and differentiation, HaCaT cells were cultured in d‐KSFM for 5 days to maintain the primary keratinocyte state and then treated with IL‐6 for 0, 1, 2, and 3 days. Compared with PBS treatment, IL‐6‐treated HaCaT cells formed tight junctions and presented a cobblestone‐like morphology (Figure 3A). EdU incorporation assays were performed to determine the effect of IL‐6 on keratinocyte proliferation, the gating strategy showed in Figure S1, the percentage of EdU‐positive cells was used for analysis of cell proliferation. The results showed that keratinocyte growth increased after exposure to IL‐6 versus PBS (Figure 3B). Furthermore, both immunoblotting and immunofluorescence staining analyses were performed to detect molecular markers of differentiated epidermal cells. Involucrin, keratin 5, and keratin 1 levels were markedly increased in HaCaT cells after exposure to IL‐6 consistent with upregulated LMO4 expression (Figure 3C,D).

**Figure 4:**
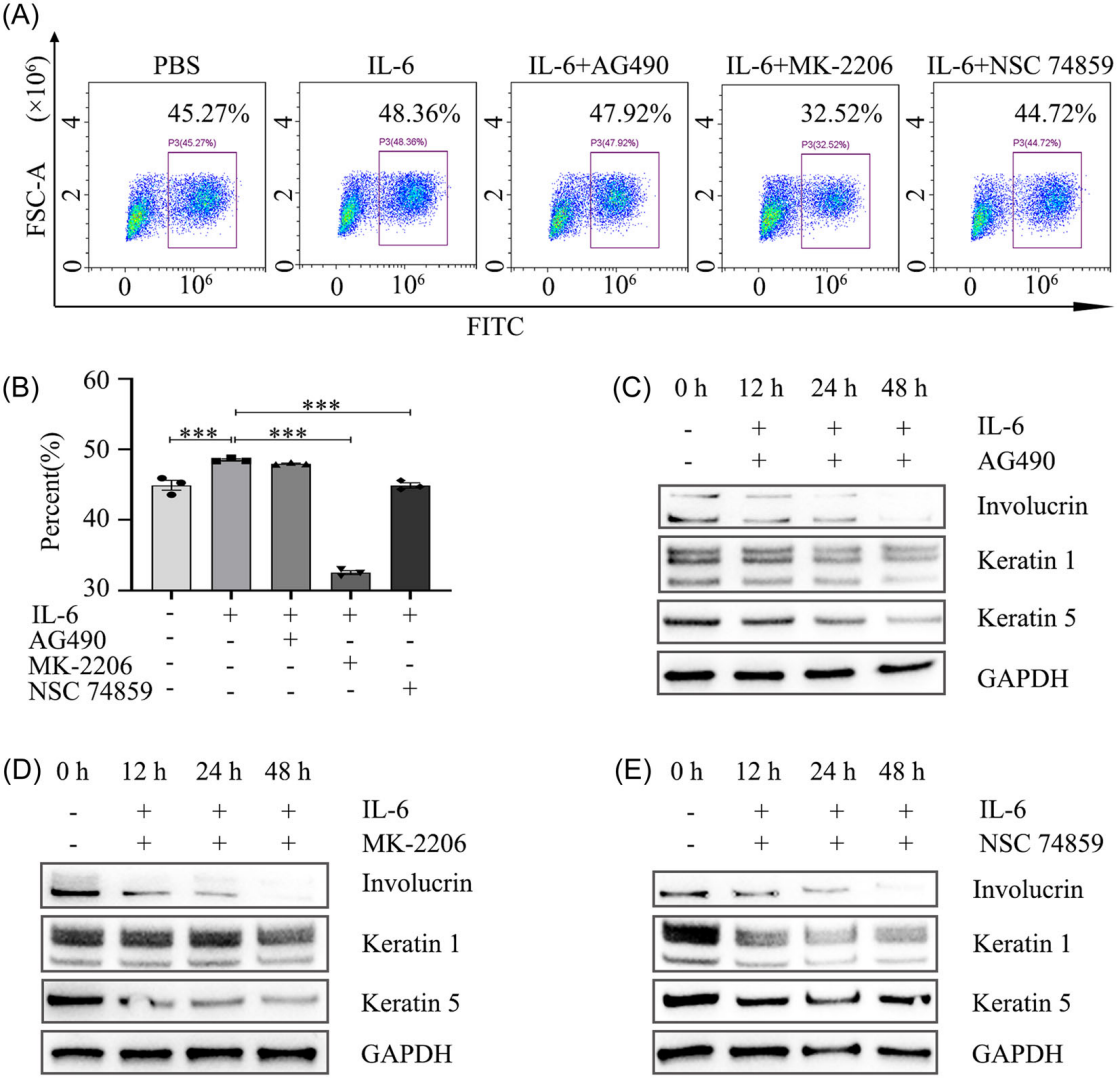
Inhibiting IL‐6‐activated the AKT/STAT3 signaling pathway weakens keratinocytes growth and differentiation. (A, B) EdU incorporation analysis of the growth of HaCaT cells treated with PBS, IL‐6 and IL‐6 with different inhibitors for 24 h. The data represent the mean ± SEM of three independent experiments. ***p < .001. AG490 (10 μM), a JAK2 inhibitor. MK‐2206 (10 μM), an AKT inhibitor. NSC 74859 (80 μM), a STAT3 inhibitor. (C), (D), and (E) Immunoblotting analysis of differentiation markers involucrin, keratin 1, and keratin 5 in HaCaT cells treated for 0, 12, 24, or 48 h with IL‐6 and AG490, MK‐2206, and NSC 74859, respectively.

To further investigate whether keratinocyte proliferation and differentiation is induced by AKT/STAT3 signaling pathway activation, inhibitors were used to block the AKT/STAT3 signaling pathway activated by IL‐6. The EdU incorporation assay showed that JAK2, AKT, and STAT3 inhibitors attenuated cell growth compared to IL‐6 treatment (Figure 4A,B). As shown in Figure 4C, IL‐6‐induced involucrin, keratin1, and keratin 5 expressions dramatically decreased in keratinocytes after exposure to JAK2 inhibitors. Notably, both the STAT3 inhibitor (NSC 74859) and the AKT inhibitor (MK‐2206) significantly decreased the expression of involucrin, keratin1, and keratin 5 (Figure 4D,E).

## 4 DISCUSSION

LMO4 is a transcription factor that regulates the proliferation and differentiation of various cells such as neurons, retinal cells, and mammary epithelial cells.^13^, ^40^, ^41^ LMO4 interacts with LDB1 to regulate normal epithelial cell differentiation and proliferation.^42^, ^43^ However, in the mammary epithelium, the dysregulation of LMO4 causes mammary neoplasia by altering cell proliferation, differentiation, and invasion.^42^, ^44^ During embryonic development, LMO4 is also expressed in keratinocytes in the basal layers of the epidermis, and interacts with Get‐1 to regulate keratinocyte differentiation and proliferation.^13^, ^45^, ^46^, ^47^ We demonstrated that LMO4 overexpression in psoriatic lesions is involved in the abnormal differentiation and hyperplasia of epidermal keratinocytes.^16^ Consistent with LMO4 expression, IL‐23 expression significantly increased in psoriatic lesions. IL‐23 is a key cytokine in the pathogenesis of psoriasis and mainly involved in Th17 cell development.^20^, ^48^, ^49^ However, IL‐23‐induced LMO4 expression plays a critical role in regulating keratinocyte proliferation and differentiation. Furthermore, LMO4 overexpression in psoriatic keratinocytes is required for IL‐23 to activate the AKT/STAT3 signaling pathway.^16^

IL‐6 is another important member of the cytokine network that regulates keratinocyte proliferation and differentiation in psoriasis.^20^, ^35^, ^50^ Although there is no correlation between IL‐6 level and disease activity, studies have confirmed that patients with psoriasis have significantly increased serum IL‐6 levels and overexpression in psoriatic lesions versus healthy controls.^51^, ^52^ In this study, we found that plasma IL‐6 levels were higher in patients with psoriasis than in healthy populations and were abnormally expressed in psoriatic lesions and imiquimod‐induced psoriasis‐like lesions. Interestingly, IL‐6 expression was significantly increased in psoriatic lesions consistent with LMO4 overexpression in the present study. We investigated the association between LMO4 overexpression and elevated levels of IL‐6. We found that IL‐6 induced LMO4 expression in HaCaT cells in a time‐dependent manner. The IHC analysis indicated that the AKT/STAT3 signaling pathway is activated in both human psoriatic lesions and mouse psoriasis‐like skin. STAT3, an important regulator of inflammation, is recognized as the main mediator of IL‐6 function and is activated in psoriatic skin.^5^, ^53^ We previously identified that p‐STAT3 regulates LMO4 expression by binding to a specific motif in the promotion of LMO4.^16^ Our in vitro experiments further demonstrated that IL‐6 induced LMO4 overexpression in HaCaT cells through the AKT/STAT3 signaling pathway activation.

IL‐23‐induced LMO4 highexpression plays a critical roles in regulating keratinocyte proliferation and differentiation, here we show that IL‐6 has a function similar to IL‐23. IL‐6‐induced LMO4 overexpression accelerated cell proliferation and increased expression of keratin‐1, keratin‐5, keratin‐10, and involucrin in HaCaT cells. Remarkably, both cell growth and differentiation were weakened when the IL‐6‐activated AKT/STAT3 pathway was inhibited. Thus, high IL‐6 levels activate the AKT/STAT3 signaling pathway to increase LMO4 expression and contribute to psoriatic keratinocyte growth and differentiation. The abnormal proliferation and differentiation of epidermal keratinocytes is an important link in the development of psoriasis.

Together, we found that in addition to IL‐23, IL‐6 also induces LMO4 overexpression in epidermal keratinocytes. IL‐6‐activated AKT/STAT3 signaling induces LMO4 expression, leading to keratinocyte hyperproliferation and mis‐differentiation. This study provides new insight into the role of LMO4 in the development of psoriasis.

## AUTHOR CONTRIBUTIONS

*Conception and design*: Haisheng Zhou, Siping Zhang, and Zhenzhen Tu. *Administrative support*: Haisheng Zhou and Siping Zhang. *Provision of study materials or patients*: Haisheng Zhou, Qiantong Xiang, Wei Wei, and Siping Zhang. *Collection and assembly of data*: Zhenzhen Tu, Wei Wei, Qiantong Xiang, and Wenwen Wang. *Data analysis and interpretation*: Zhenzhen Tu, Wei Wei, Wenwen Wang, Siping Zhang, and Haisheng Zhou. *Manuscript writing*: Zhenzhen Tu and Haisheng Zhou. *Final approval of manuscript*: All authors.

## CONFLICT OF INTEREST STATEMENT

The authors declare no conflicts of interest.

## Supporting information

Figure S1: The gating strategy for EdU incorporation assay.

## Data Availability

The datasets used and/or analyzed during the current study are available from the corresponding author upon reasonable request.

## References

[1] Yang J , Wang Z , Zhang X . GLP‐1 receptor agonist impairs keratinocytes inflammatory signals by activating AMPK. Exp Mol Pathol. 2019;107:124‐128. 30776356 10.1016/j.yexmp.2019.01.014

[2] Shakshouk H , Erickson LA , Johnson EF , Lehman JS . Updates and proposed diagnostic approach to psoriasiform dermatoses. Adv Anat Pathol. 2022;29(5):263‐274. 35180737 10.1097/PAP.0000000000000333

[3] Camporro ÁF , Roncero‐Riesco M , Revelles‐Peñas L , et al. The ñ sign: a visual clue for the histopathologic diagnosis of psoriasis. JAMA Dermato. 2022;158(4):451‐452. 10.1001/jamadermatol.2022.0015 PMC8867387 35195665

[4] Ma Y , Kim BH , Yun SK , Roh YS . *Centipeda minima* extract inhibits inflammation and cell proliferation by regulating JAK/STAT signaling in macrophages and keratinocytes. Molecules. 2023;28(4):1723. 36838711 10.3390/molecules28041723 PMC9963638

[5] Miao X , Xiang Y , Mao W , Chen Y , Li Q , Fan B . TRIM27 promotes IL‐6‐induced proliferation and inflammation factor production by activating STAT3 signaling in HaCaT cells. Am J Physiol Cell Physiol. 2020;318(2):C272‐C281. 31747314 10.1152/ajpcell.00314.2019

[6] Chen XY , Xu F , Chen JQ , et al. UBE2L3 reduces TRIM21 expression and IL‐1β secretion in epidermal keratinocytes and improves psoriasis‐like skin. J Invest Dermatol. 2023;143(5):822‐831. 36502938 10.1016/j.jid.2022.10.016

[7] Zhang J , Shu J , Sun H , et al. CCN1 upregulates IL‐36 via AKT/NF‐κBandERK/CEBP β‐mediated signaling pathways in psoriasis‐like models. J Dermatol. 2023;50(3):337‐348. 36376243 10.1111/1346-8138.16611

[8] Miyai M , Hamada M , Moriguchi T , et al. Transcription factor MafB coordinates epidermal keratinocyte differentiation. J Invest Dermatol. 2016;136(9):1848‐1857. 27208706 10.1016/j.jid.2016.05.088

[9] Kanda N , Shimizu T , Tada Y , Watanabe S . IL‐18 enhances IFN‐γ‐induced production of CXCL9, CXCL10, and CXCL11 in human keratinocytes. Eur J Immunol. 2007;37(2):338‐350. 17274000 10.1002/eji.200636420

[10] Sun P , Vu R , Dragan M , et al. OVOL1 regulates psoriasis‐Like skin inflammation and epidermal hyperplasia. J Invest Dermatol. 2021;141(6):1542‐1552. 33333123 10.1016/j.jid.2020.10.025 PMC8532526

[11] Dragan M , Sun P , Chen Z , et al. Epidermis‐intrinsic transcription factor Ovol1 coordinately regulates barrier maintenance and neutrophil accumulation in Psoriasis‐Like inflammation. J Invest Dermatol. 2022;142(3 Pt A):583‐593. 34461129 10.1016/j.jid.2021.08.397 PMC9968377

[12] Masalha M , Ben‐Dov IZ , Ram O , et al. H3K27Ac modification and gene expression in psoriasis. J Dermatol Sci. 2021;103(2):93‐100. 34281744 10.1016/j.jdermsci.2021.07.003

[13] Kenny DA , Jurata LW , Saga Y , Gill GN . Identification and characterization of LMO4, an LMO gene with a novel pattern of expression during embryogenesis. Proc Nat Acad Sci. 1998;95(19):11257‐11262. 9736723 10.1073/pnas.95.19.11257 PMC21629

[14] Kudryavtseva EI , Sugihara TM , Wang N , et al. Identification and characterization of grainy head‐like epithelial transactivator (GET‐1), a novel mammalian grainy head‐like factor. Dev Dyn. 2003;226(4):604‐617. 12666198 10.1002/dvdy.10255

[15] Sum EYM , O'Reilly LA , Jonas N , Lindeman GJ , Visvader JE . The LIM domain protein Lmo4 is highly expressed in proliferating mouse epithelial tissues. J Histochem Cytochem. 2005;53(4):475‐486. 15805422 10.1369/jhc.4A6553.2005

[16] Tu Z , Zhang S , Zhou G , et al. LMO4 is a Disease‐provocative transcription coregulator activated by IL‐23 in psoriatic keratinocytes. J Invest Dermatol. 2018;138(5):1078‐1087. 29258893 10.1016/j.jid.2017.12.010

[17] Goldie SJ , Cottle DL , Tan FH , et al. Loss of GRHL3 leads to TARC/CCL17‐mediated keratinocyte proliferation in the epidermis. Cell Death Dis. 2018;9(11):1072. 30341279 10.1038/s41419-018-0901-6 PMC6195598

[18] Chen X , Lloyd SM , Kweon J , Gamalong GM , Bao X . Epidermal progenitors suppress GRHL3‐mediated differentiation through intronic polyadenylation promoted by CPSF‐HNRNPA3 collaboration. Nat Commun. 2021;12(1):448. 33469008 10.1038/s41467-020-20674-3 PMC7815847

[19] Parisi L , Mockenhaupt C , Rihs S , Mansour F , Katsaros C , Degen M . Consistent downregulation of the cleft lip/palate‐associated genes IRF6 and GRHL3 in carcinomas. Front Oncol. 2022;12:1023072. 36457487 10.3389/fonc.2022.1023072 PMC9706198

[20] de Alcantara CC , Reiche EMV , Simao ANC . Cytokines in psoriasis. Adv Clin Chem. 2021;100:171‐204. 33453865 10.1016/bs.acc.2020.04.004

[21] Singh R , Koppu S , Perche PO , Feldman SR . The cytokine mediated molecular pathophysiology of psoriasis and its clinical implications. Int J Mol Sci. 2021;22(23):12793. 34884596 10.3390/ijms222312793 PMC8657643

[22] Sachen KL , Arnold Greving CN , Towne JE . Role of IL‐36 cytokines in psoriasis and other inflammatory skin conditions. Cytokine. 2022;156:155897. 35679693 10.1016/j.cyto.2022.155897

[23] Mellor LF , Gago‐Lopez N , Bakiri L , et al. Keratinocyte‐derived S100A9 modulates neutrophil infiltration and affects psoriasis‐like skin and joint disease. Ann Rheum Dis. 2022;81(10):1400‐1408. 35788494 10.1136/annrheumdis-2022-222229 PMC9484400

[24] Fukaura R , Akiyama M . Targeting IL‐36 in inflammatory skin diseases. BioDrugs. 2023;37(3):279‐293. 36867370 10.1007/s40259-023-00587-5

[25] Cuesta‐Gomez N , Medina‐Ruiz L , Graham GJ , et al. IL‐6 and TGF‐beta‐secreting adoptively‐transferred murine mesenchymal stromal cells accelerate healing of psoriasis‐like skin inflammation and upregulate IL‐17A and TGF‐beta. Int J Mol Sci. 2023;24(12):10132. 10.3390/ijms241210132 37373278 PMC10298958

[26] Zhu N , Guan H , Wang X , et al. EZH2 promotes angiogenesis in peritoneal dialysis by epigenetically activating SP4 expression in the IL‐6/sIL‐6R signalling pathway. Int J Med Sci. 2023;20(1):114‐124. 36619221 10.7150/ijms.78428 PMC9812808

[27] Zhu W , Liu YQ , Liu P , Cao J , Shen AG , Chu PK . Blood‐glucose‐depleting hydrogel dressing as an activatable photothermal/chemodynamic antibacterial agent for healing diabetic wounds. ACS Appl Mater Interfaces. 2023;15(20):24162‐24174. 37166230 10.1021/acsami.3c03786

[28] Xiao R , Lei C , Zhang Y , Zhang M . Interleukin‐6 in retinal diseases: from pathogenesis to therapy. Exp Eye Res. 2023;233:109556. 37385535 10.1016/j.exer.2023.109556

[29] Li Y , Jia Y , Cui T , Zhang J . IL‐6/STAT3 signaling pathway regulates the proliferation and damage of intestinal epithelial cells in patients with ulcerative colitis via H3K27ac. Exp Ther Med. 2021;22(2):890. 34194568 10.3892/etm.2021.10322 PMC8237277

[30] Tanaka T , Narazaki M , Kishimoto T . Interleukin (IL‐6) immunotherapy. Cold Spring Harbor Perspect Biol. 2018;10(8):a028456. 10.1101/cshperspect.a028456 PMC6071487 28778870

[31] Kaur S , Bansal Y , Kumar R , Bansal G . A panoramic review of IL‐6: structure, pathophysiological roles and inhibitors. Bioorg Med Chem. 2020;28(5):115327. 31992476 10.1016/j.bmc.2020.115327

[32] Zhao Y , Luan H , Jiang H , et al. Gegen qinlian decoction relieved DSS‐induced ulcerative colitis in mice by modulating Th17/Treg cell homeostasis via suppressing IL‐6/JAK2/STAT3 signaling. Phytomedicine. 2021;84:153519. 33640781 10.1016/j.phymed.2021.153519

[33] Ghosh R , Dey R , Sawoo R , Haque W , Bishayi B . Endogenous neutralization of TGF‐β and IL‐6 ameliorates septic arthritis by altering RANKL/OPG interaction in lymphocytes. Mol Immunol. 2022;152:183‐206. 36371814 10.1016/j.molimm.2022.10.015

[34] Schumertl T , Lokau J , Rose‐John S , Garbers C . Function and proteolytic generation of the soluble interleukin‐6 receptor in health and disease. Biochim Biophys Act Mol Cell Res. 2022;1869(1):119143. 10.1016/j.bbamcr.2021.119143 34626681

[35] Xu H , Liu J , Niu M , et al. Soluble IL‐6R‐mediated IL‐6 trans‐signaling activation contributes to the pathological development of psoriasis. J Mol Med. 2021;99(7):1009‐1020. 33835216 10.1007/s00109-021-02073-3

[36] Guo J , Qi C , Liu Y , et al. Terrestrosin D ameliorates skin lesions in an imiquimod‐induced psoriasis‐like murine model by inhibiting the interaction between substance P and dendritic cells. Phytomedicine. 2022;95:153864. 34923236 10.1016/j.phymed.2021.153864

[37] Singh VK , Sahoo D , Agrahari K , et al. Anti‐inflammatory, anti‐proliferative and anti‐psoriatic potential of apigenin in RAW 264.7 cells, HaCaT cells and psoriasis like dermatitis in BALB/c mice. Life Sci. 2023;328:121909. 37414141 10.1016/j.lfs.2023.121909

[38] Goruppi S , Clocchiatti A , Bottoni G , et al. The ULK3 kinase is a determinant of keratinocyte self‐renewal and tumorigenesis targeting the arginine methylome. Nat Commun. 2023;14(1):887. 36797248 10.1038/s41467-023-36410-6 PMC9935893

[39] Ochsner SA , Pedroza M , Pillich RT , et al. IL17A blockade with ixekizumab suppresses MuvB signaling in clinical psoriasis. J Invest Dermatol. 2023;143(9):1689‐1699. 36967086 10.1016/j.jid.2023.03.1658

[40] Jin K , Xiao D , Andersen B , Xiang M . Lmo4 and other LIM domain only factors are necessary and sufficient for multiple retinal cell type development. Dev Neurobiol. 2016;76(8):900‐915. 26579872 10.1002/dneu.22365

[41] Ma X , Zhang H , Li Q , et al. FOXM1 promotes head and neck squamous cell carcinoma via activation of the Linc‐ROR/LMO4/AKT/PI3K axis. Front Oncol. 2021;11:658712. 34447693 10.3389/fonc.2021.658712 PMC8383294

[42] Simonik EA , Cai Y , Kimmelshue KN , et al. LIM‐only protein 4 (LMO4) and LIM domain binding protein 1 (LDB1) promote growth and metastasis of human head and neck cancer (LMO4 and LDB1 in Head and Neck Cancer). PLoS One. 2016;11(10):e0164804. 27780223 10.1371/journal.pone.0164804 PMC5079595

[43] Singh N , Singh D , Modi D . LIM homeodomain (LIM‐HD) genes and their co‐regulators in developing reproductive system and disorders of sex development. Sex Dev. 2022;16(2‐3):147‐161. 34518474 10.1159/000518323

[44] Ding K , Wu Z , Li X , Sheng Y , Wang X , Tan S . LMO4 mediates trastuzumab resistance in HER2 positive breast cancer cells. Am J Cancer Res. 2018;8(4):594‐609. 29736306 PMC5934551

[45] Yu Z , Lin KK , Bhandari A , et al. The grainyhead‐like epithelial transactivator Get‐1/Grhl3 regulates epidermal terminal differentiation and interacts functionally with LMO4. Dev Biol. 2006;299(1):122‐136. 16949565 10.1016/j.ydbio.2006.07.015

[46] Wang N , Lin KK , Lu Z , et al. The LIM‐only factor LMO4 regulates expression of the BMP7 gene through an HDAC2‐dependent mechanism, and controls cell proliferation and apoptosis of mammary epithelial cells. Oncogene. 2007;26(44):6431‐6441. 17452977 10.1038/sj.onc.1210465

[47] Hislop NR , Caddy J , Ting SB , et al. Grhl3 and Lmo4 play coordinate roles in epidermal migration. Dev Biol. 2008;321(1):263‐272. 18619436 10.1016/j.ydbio.2008.06.026

[48] Chandrasekharan UM , Kaur R , Harvey JE , et al. TNFR2 depletion reduces psoriatic inflammation in mice by downregulating specific dendritic cell populations in lymph nodes and inhibiting IL‐23/IL‐17 pathways. J Invest Dermatol. 2022;142(8):2159‐2172. 35090950 10.1016/j.jid.2021.12.036 PMC9314460

[49] Bruno M , Davidson L , Koenen HJPM , et al. Immunological effects of Anti‒IL‐17/12/23 therapy in patients with psoriasis complicated by candida infections. J Invest Dermatol. 2022;142(11):2929‐2939. 35662644 10.1016/j.jid.2022.05.1083

[50] Ravipati A , Nolan S , Alphonse M , et al. IL‐6R/signal transducer and activator of transcription 3 signaling in keratinocytes rather than in T cells induces psoriasis‐Like dermatitis in mice. J Invest Dermatol. 2022;142(4):1126‐1135. 34626614 10.1016/j.jid.2021.09.012 PMC8957489

[51] Tong N , Zhang Y , Yang A , Dai X , Hao S . The potency of common proinflammatory cytokines measurement for revealing the risk and severity of anxiety and depression in psoriasis patients. J Clin Lab Anal. 2022;36(9):e24643. 35944185 10.1002/jcla.24643 PMC9459285

[52] Wang Q , Yan D , Zheng S , et al. Cytokine profiles and the relationship of disease severity in patients with psoriasis. Indian J Dermatol. 2022;67(2):204. 10.4103/ijd.ijd_79_22 PMC9455096 36092198

[53] Kishimoto M , Komine M , Sashikawa‐Kimura M , et al. STAT3 activation in psoriasis and cancers. Diagnostics. 2021;11(10):1903. 34679602 10.3390/diagnostics11101903 PMC8534757

