# Supplementary figures and images for: Pro‐inflammatory cytokine IL‐6 regulates LMO4 expression in psoriatic keratinocytes via AKT/STAT3 pathway
Source: Immun Inflamm Dis. 2023 Dec 6;11(12):e1104. doi: 10.1002/iid3.1104 (PMC10698831; doi:10.1002/iid3.1104)

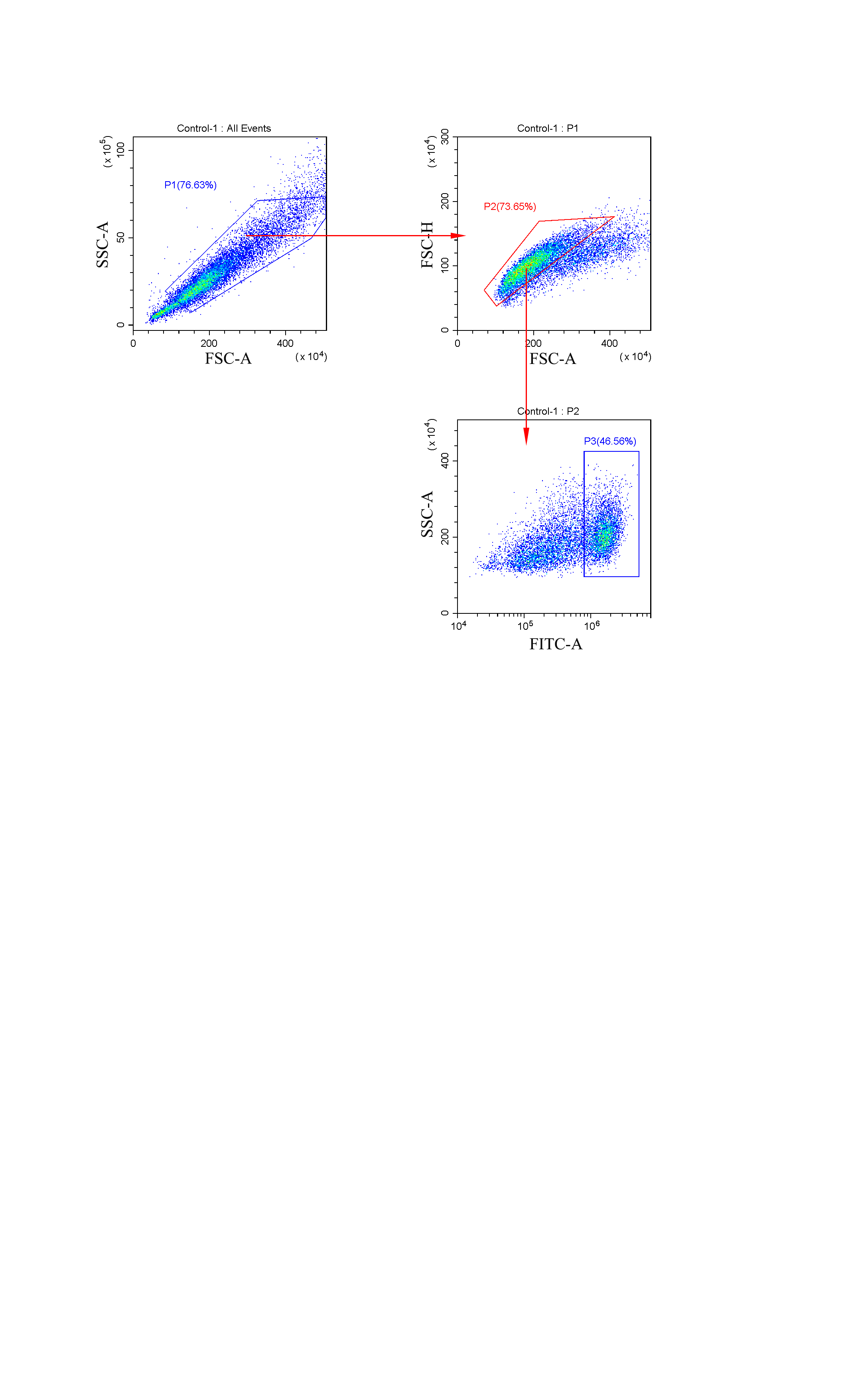

Supplement: Supplementary file 1 — Figure S1: The gating strategy for EdU incorporation assay. [file IID3-11-e1104-s001.tif]
